# Controlling oxygen coordination and valence of network forming cations

**DOI:** 10.1038/s41598-020-63786-y

**Published:** 2020-04-28

**Authors:** Takuya Aoyagi, Shinji Kohara, Takashi Naito, Yohei Onodera, Motomune Kodama, Taigo Onodera, Daiko Takamatsu, Shuta Tahara, Osami Sakata, Tatsuya Miyake, Kentaro Suzuya, Koji Ohara, Takeshi Usuki, Yamato Hayashi, Hirotsugu Takizawa

**Affiliations:** 1 Hitachi Research Laboratory, Hitachi Ltd., 7-1-1 Omika, Hitachi, Ibaraki 319-1292 Japan; 2 Tohoku University, 6-6-07 Aoba-yama, Sendai, Miyagi 980-8579 Japan; 3 Light/Quantum Beam Field, Research Center for Advanced Measurement and Characterization, National Institute for Material Science (NIMS), 1-1-1 Kouto, Sayo-cho, Sayo-gun, Hyogo 679-5148 Japan; 4 Center for Materials Research by Information Integration (CMI2) Research and Services Division of Materials Data and Integrated System (MaDIS), NIMS, 1-2-1 Sengen, Tsukuba, Ibaraki 305-0047 Japan; 5 PRESTO, Japan Science and Technology Agency, 7 Gobancho, Chiyoda-ku, Tokyo 102-0076 Japan; 6 Diffraction and Scattering Division, Japan Synchrotron Radiation Research Institute/SPring-8, 1-1-1 Kouto, Sayo-cho, Sayo-gun, Hyogo 679-5198 Japan; 7 Institute for Integrated Radiation and Nuclear Science, Kyoto University, 2-1010 Asashiro-nishi, Kumatori-cho, Sennan-gun, Osaka 590-0494 Japan; 8 University of the Ryukyus, 1 Senbaru, Nishihara-cho, Okinawa 903-0213 Japan; 9 Japan Atomic Energy Agency/J-PARC, 2-4 Shirakata, Tokai-mura, Naka-gun, Ibaraki 319-1195 Japan; 10 Yamagata University, 1-4-12 Kojirakawa-machi, Yamagata-shi, 990-8560 Japan

**Keywords:** Structure of solids and liquids, Atomistic models

## Abstract

Understanding the structure-property relationship of glass material is still challenging due to a lack of periodicity in disordered materials. Here, we report the properties and atomic structure of vanadium phosphate glasses characterized by reverse Monte Carlo modelling based on neutron/synchrotron X-ray diffraction and EXAFS data, supplemented by Raman and NMR spectroscopy. In vanadium-rich glass, the water durability, thermal stability and hardness improve as the amount of P_2_O_5_ increases, and the network former of the glass changes from VO_*x*_ polyhedra to the interplay between VO_*x*_ polyhedra and PO_4_ tetrahedra. We find for the first time that the coordination number of oxygen atoms around a V^4+^ is four, which is an unusually small coordination number, and plays an important role for water durability, thermal stability and hardness. Furthermore, we show that the similarity between glass and crystal beyond the nearest neighbour distance is important for glass properties. These results demonstrate that controlling the oxygen coordination and valence of the network-forming cation is necessary for designing the properties of glass.

## Introduction

Oxide glass components are basically classified into network formers, network modifiers and intermediates by Zachariasen^1^ and Sun^2^. Typical network formers satisfy Zachariasen’s rules^1^, but V_2_O_5_ is classified as a network former^1^ or intermediate^3^. This is because it is hard for this oxide to form glass on its own because the oxygen coordination number is five in a crystalline phase, which is larger than the coordination number of typical network formers of three and four. V_2_O_5_ based glasses have been widely studied for decades because V_2_O_5_ glass is a typical semiconducting glass originating from hopping conduction. Indeed, not only fundamental research on electrical properties^4–8^ but also applied research on cathode materials for lithium, sodium and magnesium ion batteries have been reported^9–12^. This glass also has a low glass transition temperature and relatively low thermal expansion^4^. These are quite attractive properties for low-melting glass used for sealing. In this field, lead borate glass with a high percentage of PbO has been applied to sealing below 400 °C in electronic devices, such as IC ceramic packages, crystal oscillators and micro-electro-mechanical systems. Since lead components are a hazardous substance for human health and the environment, it is necessary to avoid the use of PbO, and hence, V_2_O_5_ based glass is a promising material for overcoming this problem^13–19^. However, there are two critical problems that face the practical application of V_2_O_5_ based glass: poor water durability and low thermal stability.

Several components, i.e., P_2_O_5_, TeO_2_, GeO_2_, BaO and PbO, can vitrify V_2_O_5_ in binary systems^4^. Above all, the most common vitrification component is P_2_O_5_ because the V_2_O_5_-P_2_O_5_ system has the widest range of vitrification in binary systems. Moreover, the properties of this binary system change drastically with the amount of P_2_O_5_ compared with other components^20,21^. For example, the glass transition temperature increases about 200 °C, the electrical resistivity increases to the double-digit range, and water resistance improves from water soluble to nearly insoluble in the composition range from 80V_2_O_5_-20P_2_O_5_ to 50V_2_O_5_-50P_2_O_5_^20,21^. Conversely, we can greatly design the properties of V_2_O_5_ based glass by controlling the amount of P_2_O_5_ content. To design compatible properties for various applications, understanding the glass structure is crucial. Structures of V_2_O_5_ glass and (100-*x*)V_2_O_5_-*x*P_2_O_5_ (VP*x*) glasses have been widely studied by using spectrometry techniques^22,23^ and diffraction techniques^24–26^. The results for VP*x* glasses obtained by using a Fourier transform infrared spectrometer (FT-IR) indicate a structural change from V_2_O_5_- to β-VOPO_4_-like structures around *x* = ~25^22^. The valence states and structure model of *x* = 40–70 glass obtained from X-ray photoelectron spectroscopy (XPS) data suggested that this model consisted of a mixture of vanadate phosphate phases, including V_2_O_5_, VOPO_4_, (VO)_2_P_2_O_7_, VO(PO_3_) and V(PO_3_)_3_^23^. X-ray and neutron diffraction measurements conclude that V-O structural units undergo complex changes as the amount of V_2_O_5_ content decreases^25^. According to an analysis, short-range structural units change from a VO_4+1_ trigonal pyramid for *x* = 0, VO_4+1_ trigonal bipyramid and VO_5+1_ square pyramid for *x* = 27 and VO_5+1_ square pyramid for *x* = 27–50 to a VO_5+1_ square pyramid of β-VOPO_4_ and VO_6_ distorted octahedron of (VO)_2_P_2_O_7_ for *x* = 50. In addition, the average V-O coordination number (*N*_V-O_) for VP*x* glasses changes from 4.5 (*x* = 10) to 5.1 (*x* = 50) as the amount of V_2_O_5_ content decreases^25^.

Nevertheless, the structure-property relationship in VP*x* glass is still unclear; in particular, the water durability, thermal stability and hardness of this glass are important properties for practical application. One reason is that the coordination environment and valence of vanadium atoms are still unknown. The other is that the nature of network formation in the glass still has not been well revealed. In this article, we report on the properties and atomic structures of VP*x* glasses characterized by reverse Monte Carlo (RMC) modelling based on neutron/synchrotron X-ray diffraction data and extended X-ray absorption fine structure (EXAFS) data measured at the V-*K* absorption edge. In addition, we used Raman and ^51^V magic angle spinning (MAS) nuclear magnetic resonance (NMR) spectroscopy to determine the connectivity of PO_4_ tetrahedra and the coordination number of pentavalent VO_*x*_ polyhedra, respectively. On the basis of state-of-the art experimental data aided by a data-driven modelling technique, we discuss the relationship between several properties and glass structures with a special focus on the coordination environment and valence of vanadium atoms.

## Results

### Glass properties

Glass compositions, the ratio of V^4+^/V_total_, densities and melting temperatures of VP*x* glass samples are summarized in Table 1. The V^4+^/V_total_ ratio of the prepared samples increased as the amount of P_2_O_5_ increased. This trend is supported by the shift in the energy of the absorption edge of the X-ray absorption near-edge spectroscopy (XANES) spectra at the V-*K* edge (Fig. S1) and is consistent with the results of a previous study^20^. However, the values of the previous study differed from those of this study^22^ because the results were affected greatly by the melting conditions.

**Table 1:** Composition and properties of VP*x* glasses.

Sample No.	Composition /mol% ± 0.5		[V^4+^]/[V_total_] ± 0.02	Density/g cm^−3^ ± 0.0004	Atomic number density/atom Å^−3^	Melting temperature/°C	Thermal stability/°C ± 2		
	V_2_O_5_	P_2_O_5_					*T*_g_	*T*_c_	Δ*T*
VP10	90.2	9.8	0.06	2.9712	0.0702	900	224	253	29
VP19	81.0	19.0	0.14	2.9315	0.0705	950	244	379	135
VP28	71.5	28.5	0.24	2.8926	0.0709	1000	280	420	140
VP37	63.5	37.5	0.38	2.8691	0.0716	1000	348	545	197
VP44	55.8	44.2	0.57	2.8613	0.0723	1150	413	None	None

**Figure 1:**
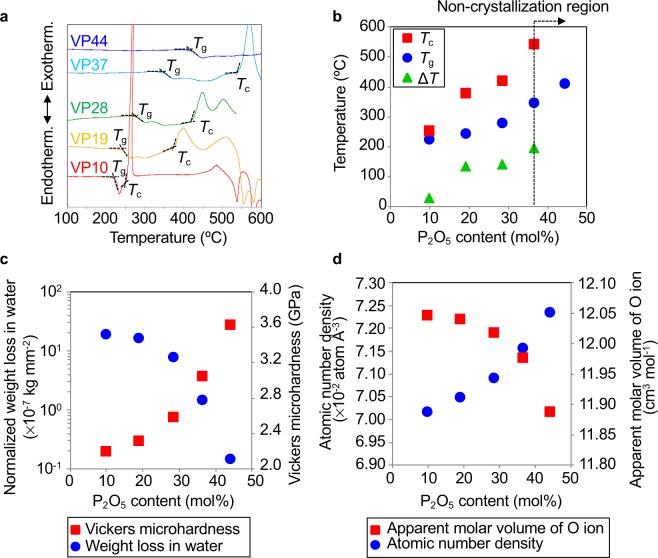
Characterization of VP*x* glasses. (**a**) Differential thermal analysis curves. (**b**) Glass transition temperatures and crystallization temperatures. (**c**) Normalized weight loss in water and Vickers microhardness. (**d**) Atomic number densities and apparent molar volume of oxygen ion. In VP44 glass, no crystallization peak was observed until glass melted.

The glass transition temperature *T*_g_ and crystallization temperature *T*_c_ determined from differential thermal analysis (DTA) curves of these samples are shown in Fig. 1a,b and are summarized in Table 1. It is demonstrated from Fig. 1b that these characteristic temperatures and thermal stability ∆*T* = *T*_c_ − *T*_g_ increased as the P_2_O_5_ amount increased, that P_2_O_5_ is an element for improving thermal stability in the V_2_O_5_ glass system. In particular, no crystallization peak was observed in the VP44 glass, and the glass softened and flowed to the melting temperature without crystallization. The normalized weight loss in water and Vickers microhardness of these samples, shown in Fig. 1c, suggest that the water durability improved drastically, whilst the glass hardened as the P_2_O_5_ amount increased. The atomic number densities and apparent molar volume of O ions for these samples are shown in Fig. 1d. It was confirmed that the atomic number density increased, whereas the apparent molar volume of O ions decreased as the amount of P_2_O_5_ increased. Both indicate that the packing density of atoms increased with the addition of P_2_O_5_. Indeed, the atomic number density and apparent molar volume of O ions for crystalline V_2_O_5_ were 0.0779 Å^−3^ and 10.83 cm^3^ mol^−1^, respectively, suggesting that the packing densities of these glass samples were smaller than that of crystalline V_2_O_5_.

### Diffraction and EXAFS data

**Figure 2:**
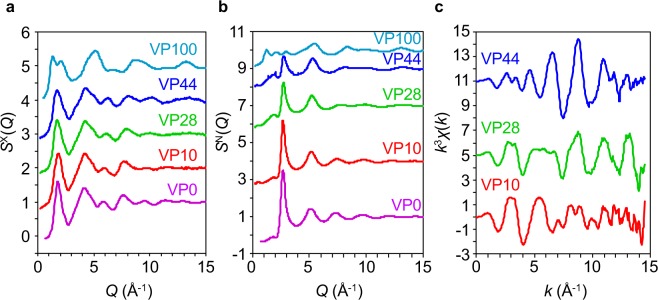
X-ray/neutron diffraction and EXAFS data in reciprocal space for VP*x* glasses. (**a**) X-ray total structure factors, *S*^X^(*Q*). (**b**) Neutron total structure factors, *S*^N^(*Q*). (**c**) EXAFS *k*^3^*χ*(*k*) measured at the V-*K* edge.

Data on X-ray structure factors, *S*^X^(*Q*), neutron total structure factors, *S*^N^(*Q*) and EXAFS *k*^3^*χ*(*k*) measured at the V-*K* edge for a series of VP*x* glasses are summarized in Fig. 2a–c, respectively. It is confirmed that all samples are homogeneous, because we cannot observe any small angle scattering at *Q* < 1 Å^−1^ in Fig. 2a,b. Three-peak structure was observed for the neutron *S*^N^(*Q*) for VP100 [glassy (*g*)-P_2_O_5_]^26^ glass, for which we can assign the peak at *Q* = 1.25 Å^−1^ and that at *Q* = 2.1 Å^−1^ to a split first sharp diffraction peak (FSDP)^27^, and the peak at *Q* = 2.95 Å^−1^ to the principal peak (PP)^27^. A split FSDP was not observed for glassy (*g*)-SiO_2_ nor glassy (*g*)-GeO_2_^28^, but it was observed for the VP100 glass due to the formation of a Q^3^ network, in which a phosphorous atom has three bridging oxygen atoms and one bridging oxygen atom. In contrast, we observed only a two-peak structure (FSDP at *Q* = 1.4 Å^−1^ and PP at *Q* = 2.1 Å^−1^) in the X-ray *S*^X^(*Q*) for the VP100 glass^29^ because a PP was not observed in the X-ray data due to small weighting factors of oxygen-related correlations for X-rays. Intriguingly, this complicated peak structure disappeared in the other VP*x* glasses, and it showed a sharp PP at *Q*  ~2.7 Å^−1^ in the neutron *S*^N^(*Q*) and a relatively sharp FSDP at *Q*  ~1.8 Å^−1^ in the X-ray *S*^X^(*Q*). The EXAFS data measured at the V-*K* edge differed substantially, suggesting that the local environment of V atoms changed with the glass composition.

**Figure 3:**
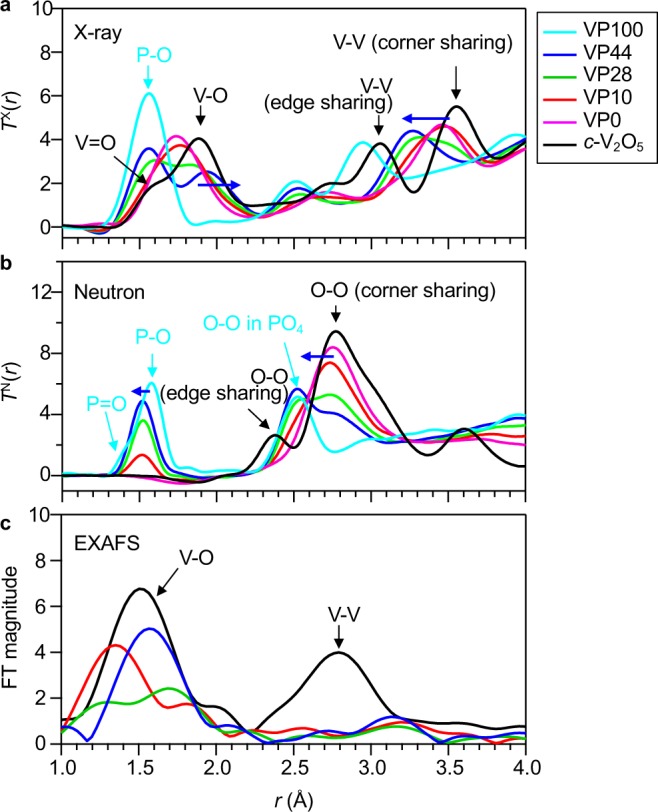
X-ray/neutron diffraction and EXAFS data in real space for VP*x* glasses together with *c*-V_2_O_5_ data. (**a**) X-ray total correlation functions, *T*^X^(*r*). **(b**) Neutron total correlation functions, *T*^N^(*r*). (**c**) Fourier-transformed (FT) EXAFS spectra for VP*x* glasses. Black, *c*-V_2_O_5_; Purple, VP0 glass^30^; Red, VP10 glass; Green, VP28 glass; Blue, VP44 glass; Cyan, VP100 glass^26,29^.

The X-ray total correlation functions, *T*^X^(*r*) and neutron total correlation functions, *T*^N^(*r*), for a series of VP*x* glasses are shown in Fig. 3a,b, respectively, together with the data for crystalline (*c*-)V_2_O_5_. We can see an excellent contrast between the X-ray and neutron diffraction data because X-rays are sensitive to vanadium, whilst oxygen can be easily detected with neutrons, and it is very difficult to detect vanadium with neutrons. In addition, it is possible to detect both P-O and V-O correlations with X-rays, as can be seen in the Fig. 3a, where a tiny negative V-O correlation peak can be observed at *r* = 1.7 Å in the neutron *T*(*r*). Indeed, only oxygen atoms can be detected with neutrons, and this allows us to observe both corner-sharing and edge-sharing O-O correlations in *c*-V_2_O_5_. The most striking feature in the X-ray *T*(*r*) is the big difference in V-O correlation peaks between the VP0 glass [glassy (*g*)-V_2_O_5_] and *c*-V_2_O_5_. In the case of *c*-V_2_O_5_, both V-O and V = O bonds were clearly distinguished, whilst such a doublet feature was not observed in the VP0 glass. Moreover, it was found that the bond length of V-O increased, whereas V-V correlations and O-O correlations in the corner sharing VO_*x*_ polyhedra, and the P-O distance decreased as the P_2_O_5_ fraction increased. These results are consistent with the results reported by Hoppe *et al*. for the VP27 and VP50 glasses^25^, indicating that the structural units of VO_*x*_ and PO_4_ polyhedra and the connectivity of VO_*x*_-VO_*x*_ and VO_*x*_-PO_4_ in each glass change with the glass composition. Figure 3c shows Fourier-transformed (FT) EXAFS spectra for the VP*x* glasses, in which significant composition-dependent modification of the local environment around the V atoms was observed in real space, too.

### Structural model of the glasses

**Figure 4:**
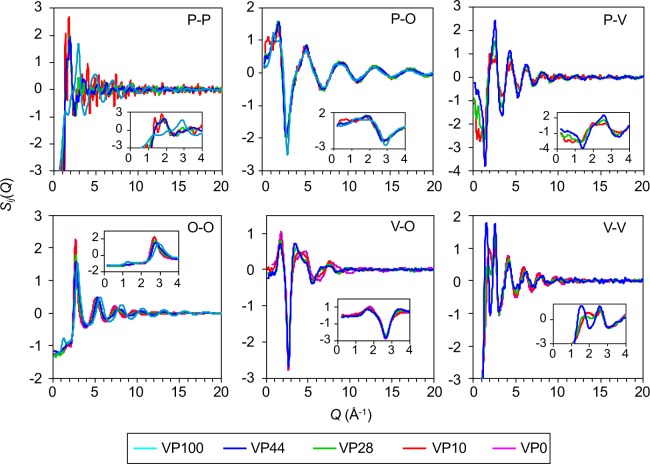
RMC-generated partial structure factors, *S*_*ij*_(*Q*), for VP*x* glasses. Purple, VP0 glass; Red, VP10 glass; Green, VP28 glass; Blue, VP44 glass; Cyan, VP100 glass^31^.

We performed RMC modelling on the basis of experimental data to uncover the relationship between glass structures and properties. As can be seen in Fig. S2, RMC-modelled X-ray *S*^X^(*Q*), neutron *S*^N^(*Q*) and V-*K* edge EXAFS *k*^3^*χ*(*k*) data for the VP0 (*g*-V_2_O_5_)^30^, VP10, VP28, VP44, and VP100 (*g*-P_2_O_5_)^31^ glasses agreed well with experimental data. Figure 4 compares the partial structure factors, *S*_*ij*_(*Q*), of a series of VP*x* glasses calculated from the RMC models. First of all, we address partial structures in the VP0 (*g*-V_2_O_5_) and VP100 (*g*-P_2_O_5_) glasses. It is well known that the FSDP of network formers, *e.g*., *g*-SiO_2_ and *g*-GeO_2_, shows up a positive peak in each *S*_*ij*_(*Q*)^28^. However, the FSDP of the *S*_PP_(*Q*) and *S*_PO_(*Q*) was a doublet^31^, and both the X-ray and neutron *S*(*Q*) did not exhibit a well-defined FSDP for VP100 glass. The reason for this is due to the different length scale that arose from both the P-O bonds (1.58 Å) and P = O bonds (1.43 Å) in the glass (see Fig. 3b), as discussed by Hoppe^26^. In comparison, a sharp positive FSDP observed at *Q*  ~1.8 Å^−1^ for three *S*_*ij*_(*Q*) in the VP0 glass, which is related to a V-O correlation peak that is relatively symmetrical in comparison with the VP0 glass. Other interesting behaviour is the very sharp negative and positive peaks observed at *Q* = 1.38 Å^−1^ and *Q* = 2.60 Å^−1^ in the *S*_P-V_(*Q*) of the VP44 glass, suggesting that the network was formed by the interplay between PO_4_ tetrahedra and VO_*x*_ polyhedra.

**Figure 5:**
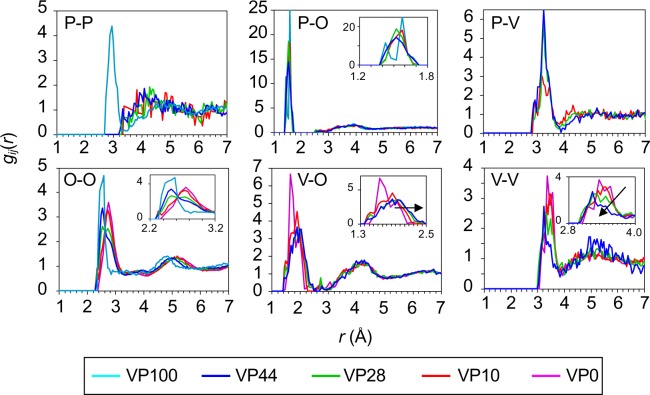
Short-range structural analysis on RMC-generated models for VP*x* glasses. Partial pair-distribution functions, *g*_*ij*_(*r*). Purple, VP0 glass; Red, VP10 glass; Green, VP28 glass; Blue, VP44 glass; Cyan, VP100 glass^31^.

Partial pair-distribution functions, *g*_*ij*_(*r*), of a series of VP*x* glasses calculated from the RMC models are shown in Fig. 5. The first P-O correlation peaks observed at *r* = 1.6 Å for the VP10, VP28 and VP44 glasses were more symmetrical in comparison with the VP100 glass, suggesting that electrons were more delocalized in the VP10, VP28 and VP44 glasses. A significant composition-dependent change was observed for *g*_O-O_(*r*), *g*_V-O_(*r*) and *g*_V-V_(*r*), and it was confirmed that this reflected the changes in the experimental *T*(*r*). The first P-V correlation peak observed at 3.2 Å became sharp as the P_2_O_5_ fraction increased, which was in line with the behaviour in *Q* space, suggesting the interplay between PO_4_ tetrahedra and VO_*x*_ polyhedra in forming the network.

**Figure 6:**
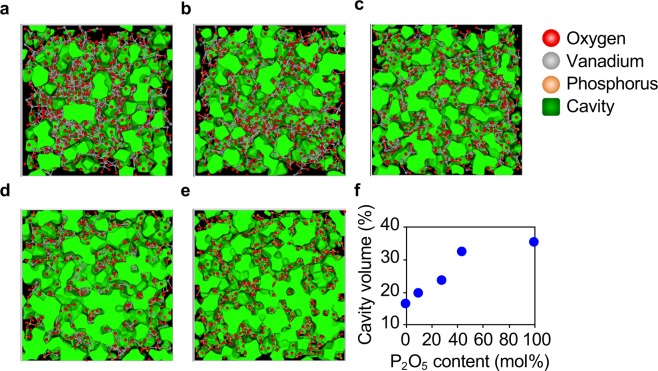
Cavity analysis. (**a**–**e**) RMC-generated atomic configurations with cavities for (**a)** VP0, (**b)** VP10, (**c)** VP28, (**d)** VP44 and (**e)** VP100 glasses. (**f)** Cavity volume as a function of P_2_O_5_ content.

The average coordination numbers calculated up to 2.5 Å and the ratio of edge-sharing VO_*x*_ polyhedra calculated from the RMC models are summarized in Table 2. The oxygen-cation coordination number *N*_O-M_ (M = V, P), the V-O coordination number *N*_V-O_ and the ratio of edge-sharing VO_*x*_ polyhedra increased as the amount of P_2_O_5_ increased. It is worth mentioning that 15% of VO_*x*_ polyhedra were edge-shared in the VP44 glass, which is outside of Zachariasen’s rule^1^. These behaviours are consistent with the fact that the packing density of atoms in glass increased as the P_2_O_5_ fraction increased. The atomic configurations together with the cavity volume of a series of VP*x* glasses are shown in Fig. 6. It is noted that the cavity volume increased as the amount of P_2_O_5_ increased, whilst packing density increased.

**Table 2:** Coordination numbers of VP*x* glasses obtained by RMC models.

Composition	Coordination numbers (*r* < 2.5 Å)						Edge-sharing rate of VO_*x*_ (%)
	*N*_P-O_	*N*_O-P_	*N*_O-O_	*N*_V-O_	*N*_O-V_	*N*_O-M_ (M = V, P)	
VP0 (V_2_O_5_)	None	None	0.27	4.43	1.77	1.77	1.1
VP10	4.00	0.16	0.66	4.46	1.64	1.80	1.5
VP28	3.99	0.48	1.21	4.52	1.40	1.88	5.0
VP44	3.98	0.80	1.43	4.75	1.22	2.02	15.0
VP100 (P_2_O_5_)	3.99	1.60	2.07	None	None	2.07	None

**Figure 7:**
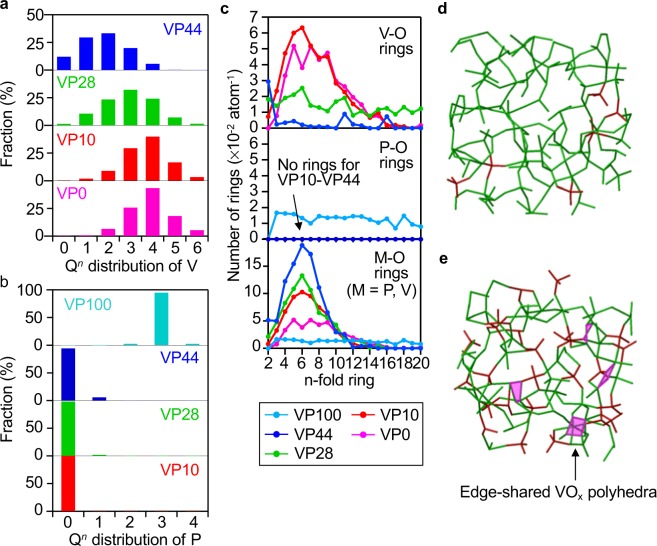
Connectivity and ring statistics of VO_*x*_ and PO_4_ polyhedra. (**a,b**) Q^*n*^ distribution of V and P species obtained by RMC modelling. (**c**) Normalized numbers of -V-O-rings, -P-O-rings and -M-O-rings (M = P, V) in VP*x* glasses. Purple, VP0 glass; Red, VP10 glass; Green, VP28 glass; Blue, VP44 glass; Cyan, VP100 glass. (**d,e**) Atomic configurations of (**d**) VP10 and (**e**) VP44 glasses are obtained from the RMC models. Green and red-coloured bond represents V-O bond and P-O bond, respectively.

The Q^*n*^ distribution of V and P species calculated from the RMC models are shown in Fig. 7a,b. Although the Q^*n*^ distribution of V species shifted to a smaller number of *n* as the amount of P_2_O_5_ increased, the V species had a high proportion at *n* ≥ 2 (Fig. 7a). In comparison, the P species were almost isolated PO_4_ (Q^0^) tetrahedra not only in the VP10 glass but also in the VP44 glass, as shown in Fig. 7b. These behaviours are supported by the Raman spectra as shown in Fig. S3, in which a peak assigned to Q^0^ was observed for the VP10 and VP44 glasses. The ring statistics of VP*x* glasses are shown in Fig. 7c. It was demonstrated that the normalized number of V-O rings decreased as the amount of P_2_O_5_ increased, whereas P-O rings did not form in the range of glasses VP10 to VP44. In contrast, the normalized number of M-O rings increased as the amount of P_2_O_5_ increased. These results strongly indicate that the network former of the VP*x* glasses changed from VO_*x*_-VO_*x*_ networks to the interplay between VO_*x*_ and PO_4_ for the VP28 and VP44 glasses. These changes in the network structure and edge-shared VO_*x*_ polyhedra were clearly visible in the three-dimensional atomic configurations obtained from the RMC models, as shown in Fig. 7d,e.

**Figure 8:**
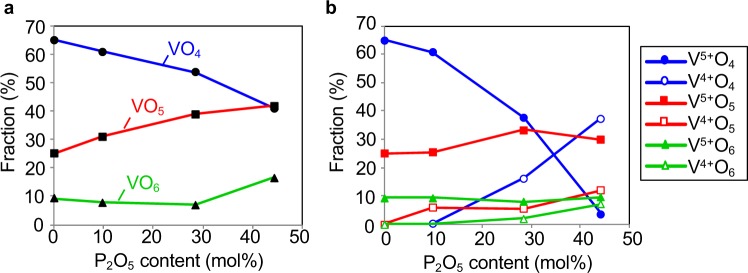
Distribution of oxygen coordination number and valence of vanadium. (**a**) Total fraction of VO_4_, VO_5_ and VO_6_ units obtained by RMC modelling. (**b**) Fraction of V^5+^O_4-6_ and V^4+^O_4-6_ units calculated by RMC modelling and ^51^V NMR spectroscopy.

The fraction of VO_*x*_ polyhedra derived from the RMC models is shown in Fig. 8a. The figure clearly shows that the majority of short-range structural units in the VP*x* glasses consisted of VO_4_ and VO_5_ units. It was also found that the fraction of VO_4_ units decreased, whilst the fraction of VO_5_ units increased systematically as the amount of P_2_O_5_ increased. Taking the vanadium valence into account, VO_*x*_ polyhedra were divided into six units, V^4+^O_4-6_ and V^5+^O_4–6_. To obtain information on the local structure around the V^5+^ in the VP*x* glasses, NMR spectroscopic measurements were performed. ^51^V NMR spectroscopy can selectively detect the diamagnetic V^5+^ state because the V^4+^ state cannot be analysed directly due to its paramagnetism. The ^51^V MAS-NMR spectra of the VP*x* glasses are shown in Fig. S4. The peaks observed at around −750, −540, −490 and −300 ppm can be assigned to symmetric-V^5+^O_4_ (*s*-VO_4_), distorted-V^5+^O_4_ (*d*-VO_4_), V^5+^O_6_ (VO_6_) and V^5+^O_5_ units (VO_5_), respectively^32–34^. The fractions of these units, as calculated from the area of each peak, are summarized in Table S1. It is suggested for the V^5+^ state in the VP*x* glasses that the fraction of V^5+^O_4_ units decreased as the amount of P_2_O_5_ increased, whereas the fraction of V^5+^O_5_ units increased. The fractions of V^5+^O_4-6_ and V^4+^O_4-6_ units calculated from the RMC models, ^51^V NMR spectroscopy and the fraction of V^4+^/V_total_ are shown in Fig. 8b. The result clearly indicates that the unusually small coordination number of V^4+^O_4_ units existed in the VP*x* glasses and the fraction increased as the amount of P_2_O_5_ increased, although it has been suggested for the V^4+^ state in glass that only V^4+^O_5_ or V^4+^O_6_ units are taken the same as crystals due to the larger ionic radius of V^4+^ ^35–38^. Such unusually small coordination numbers in oxide glass are also reported for MgO-SiO_2_^39^, CaO-Al_2_O_3_^40^ and ZnO-P_2_O_5_^41^ glass systems, and, hence, it is suggested that this may be a characteristic feature in some oxide glasses with atypical network formers.

## Discussion

We set out to model the glass structure to understand the water durability, thermal stability and hardness, which are important in practical applications. The water durability of VP*x* glasses improves as the amount of P_2_O_5_ increases, even though VP0 (*g*-V_2_O_5_) and VP100 (*g*-P_2_O_5_) glasses readily dissolve into water^31,42,43^. Gin *et al*. reported on the water durability of glass divided into several stages^44^. This improvement of VP*x* glasses in terms of water durability was determined to be due to the suppression of the hydrolysis process, which is affected by the structure of VO_*x*_ and PO_4_ polyhedra and the valence of vanadium. The cavity volume in the glass did not seem to significantly affect the water durability because, when the cavity volume increased, the water durability improved. Regarding the structure of VO_*x*_ polyhedra and valence of vanadium, Nabavi *et al*. suggested that the V^5+^O_4_ units in amorphous V_2_O_5_ are highly reactive towards water, whereas the V^5+^O_5_ units in crystalline V_2_O_5_ are not. Moreover, they also suggested that the water durability increased when the number of V^5+^O_4_ units decreased, which implies that water durability is affected by V-O coordination^35^. Our results also show that there is a clear correlation between normalized weight loss in water and the fraction of V^5+^O_4_ units in all cations (see Fig. S5). In other words, we suggest that V^4+^O_4_ units are stable against water, whilst V^5+^O_4_ units are highly reactive towards water. We conclude that this might be affected by the polarizability of VO_*x*_ polyhedra, as in the case of PO_4_ polyhedra. Therefore, the increase in edge-sharing VO_*x*_ polyhedra might improve the water durability. Regarding the structure of PO_4_ polyhedra, several papers reported that phosphate glass that has PO_4_ tetrahedra with Q^0^ or Q^1^ structures shows good water durability, suggesting that the low polarizability in PO_4_ tetrahedral units makes it hard to attract polar water molecules^45–47^. Therefore, the reason that the water durability improved in the range of glasses VP0 to VP44 is because the structures of the PO_4_ tetrahedral units were only Q^0^ and Q^1^ structures, unlike the Q^3^ structures in the VP100 glass. Hence, we conclude that the mechanism for improving water durability by mixing materials with poor water durability is the change in the structure of VO_*x*_ and PO_4_ units toward units with low polarizability that have good water durability. As reported by Feltz *et al*., if the amount of P_2_O_5_ is further increased in VP*x* glasses, the water durability decreases because the connectivities of PO_4_ tetrahedra might change into Q^2^ and/or Q^3^ structures^20^. In other words, our results imply that the water durability of VP*x* glasses can be improved by changing the interconnection of VO_*x*_ and PO_4_ polyhedra and the valence of vanadium, for example, by adding additives. This presumption is supported by the fact that the water durability of VP*x* glasses improves with the addition of Sb_2_O_3_, which causes an increase in the ratio of V^4+^/V_total_^21,48^.

The thermal stability, an indirect indicator of glass forming ability, of VP*x* glasses also improves as the amount of P_2_O_5_ increases, which indicates that the driving force of crystallization is reduced. Since a driving force arises from the difference in chemical potential between the glass state and crystalline state, our result indicates that the difference becomes smaller as the amount of P_2_O_5_ increases. We conclude that there are two reasons for this. One reason is that the atomic structure of the glasses is very far away from the conventional oxide glasses classified by Zachariasen^1^ and Sun^2^, and the similarity with a crystalline structure increases as the amount of P_2_O_5_ increases. The similarity between glass and crystal is often discussed for the fast phase-change materials used for DVD/Blu-ray media, where the topology in terms of the ring-size distribution between glass and crystal is similar, whilst coordination in the glassy phase is significantly lower than that in the crystal phase. Therefore, it is concluded that this similarity is the crucial reason for the rapid phase change between two phases^49^. Indeed, the increased packing density and large fraction of edge-sharing in the glassy state are a remarkable signature of P_2_O_5_ rich glass. Accordingly, these behaviours demonstrate that the similarity between glass and crystal beyond the nearest neighbour distance (for example, polyhedral connection and atomic packing density) is rather important for the glass properties because the first V-O coordination number and the valence of vanadium of VP*x* glasses are different from those of crystal. In other words, this indicates that the similarity beyond the nearest neighbour distance is important for decreasing the difference in chemical potential between the glassy state and crystalline state. The other reason is the high ratio of V^4+^/V_total_ in the VP*x* glasses. Sakurai *et al*. also reported that the thermal stability of VP*x* glasses improves as the amount of P_2_O_5_ increases, but that of their VP*x* glasses is significantly lower than that of our VP*x* glasses^22^. Although the packing densities of their VP*x* glasses were not mentioned in ref. ^22^, the ratio of V^4+^/V_total_ in their VP*x* glasses was lower than that of our VP*x* glasses. Accordingly, we suggest that the high ratio of V^4+^/V_total_ is an important factor for improving thermal stability, too. However, it is still not certain whether V^4+^ states themselves reduce the chemical potential between glass and crystal or that they increase the atomic packing density and edge-sharing VO_*x*_ polyhedra.

The hardness of the VP*x* glasses also improves as the amount of P_2_O_5_ increases. We conclude that this should be explained by an increased packing density associated with a higher V-O/O-M coordination number and increase in edge-sharing VO_*x*_ polyhedra. Indeed, Rosales-Sosa *et al*. reported that a high atomic packing density and dissociation energy per unit volume of components increases the values of hardness and the elastic modulus^50^. We calculated the atomic packing density in Fig. S6. It was found that the increase in P_2_O_5_ content caused the glass structure to have a higher packing density with increased V-O/O-M coordination numbers and increase in edge-shared VO_*x*_ polyhedra. The dissociation energy per unit volume of components should increase with the number of bonds per unit volume of components. Furthermore, the reduction in vanadium ions might affect the dissociation energy, whilst the cavity volume in glass should be independent of hardness because it does not affect the packing density and dissociation energy.

In addition, we have previously reported that the thermal expansion coefficients of VP*x* glasses did not change significantly despite an increased glass transition temperature as the amount of P_2_O_5_ increased^21^. Generally, the thermal expansion coefficient of glass materials increased as the glass transition temperature decreased. Such an anomalous coefficient was also identified with the network configuration in ZnO-P_2_O_5_ glass systems^41^. We calculated the fractions of M-O ring distributions as shown in Fig. S7. The distribution remained the same in the range of glasses VP10 to VP44. Hence, our results imply that this anomalous thermal expansion coefficient was related to the fractions of the distributions only because of a structural feature that does not change in the range of glasses VP10 to VP44. These results for the structure-property relationship demonstrate that the properties of glass are strongly affected by the structure and valence of network forming cations.

## Conclusion

In this article, we discussed the relationship between the properties and atomic structure of V_2_O_5_-P_2_O_5_ glass as characterized by RMC modelling on the basis of neutron and synchrotron X-ray data. The present findings indicate that the structure and valence of network formers is important for designing their properties. Adding P_2_O_5_ causes the packing density of atoms to increase and the amount of vanadium ions to decrease in V_2_O_5_-rich glass, resulting in a glass structure that is associated with an increase in edge-sharing VO_*x*_ polyhedra. We find that these are important for improving the water durability, thermal stability and hardness. In particular, the valence and structural change of vanadium affect the change in the water durability and thermal stability. We are confident that the unusually small coordination number of V^4+^ is especially important for water durability and that the similarity between glass and crystal beyond the nearest neighbour distance is important for thermal stability. The results presented in this study are a significant advance in understanding the fundamental properties of glass materials. Furthermore, this work paves the way towards glass sealing materials being completely lead-free and cathode materials for secondary batteries being improved.

## Materials and Methods

### Sample preparation

Glass samples with a nominal molar composition of (100 − *x*)V_2_O_5_-*x*P_2_O_5_ were synthesized by melt quenching of V_2_O_5_ and P_2_O_5_ powders (Kojundo Chemical Laboratory Co., Ltd.). 100 g of the mixture was melted in a platinum crucible and kept at melting temperature for one hour. The molten glass was cast onto a stainless-steel plate at 100 °C. The prepared glass samples were annealed at 10 °C higher than the glass transition temperature and slowly cooled at 1 °C min^−1^ for 40 min to relieve residual internal stress. V_2_O_5_ glass was synthesized by using a twin roller method. The oxide powder was melted at 800 °C for 5 min in a platinum crucible and then rapidly quenched by rotating rollers.

### Characterization

The cation compositions of the resulting material were determined by wavelength dispersive X-Ray fluorescence spectrometers (Rigaku, ZSX Primus II). The resulting material was fully amorphous, and this was confirmed by X-ray diffraction (XRD) using a diffractometer system equipped with a monochromatic Cu Kα radiation source (Rigaku, RINT-2000). The fraction of the reduced amount of V ion ([V^4+^]/[V_total_]) was estimated by measuring the quantities of pentavalent vanadium ions [V^5+^] and total vanadium ions [V_total_] by oxidation-reduction titration, assuming that the V ions in the glass consisted of V^5+^ and V^4+^. The densities of the glass samples were measured by using a dry pycnometer (Micromeritics, AccuPyc II 1340). The atomic number densities of these samples were calculated from the density and V^4+^/V_total_ ratio. The apparent molar volumes of O ions were also calculated from the density and V^4+^/V_total_ ratio by using the formula described by Drake *et al*.^51^. The glass transition temperature (*T*_g_) and crystallization temperature (*T*_c_) were measured by using differential thermal analysis (DTA) (Advance Riko, DT-1500) at a heat rate of 5 °C min^−1^. The weight loss in water at 70 °C for 30 min for the glass sample plates was measured to determine the water resistance. Vickers microhardness measurements were made on the sample surfaces at room temperature by using an auto hardness test system (Matsuzawa, AMT-X7FS) with an accuracy of ±0.04 GPa. A load was applied for 0.98 N, 15 s.

### Structural analysis of glass

High-energy X-ray diffraction experiments (HEXRD) were carried out at room temperature by using the BL04B2 beamline of SPring-8^52^. The incident X-ray energy was 61.6 keV as obtained from a Si(220) crystal monochromator. Diffraction patterns of the samples, placed in a silica glass tube, were measured in a transmission geometry. The collected data were corrected by using a standard program^52^. X-ray absorption fine structure (XAFS) experiments were carried out by using the BL14B2 beamline of SPring-8^53^. The XAFS samples were ground with boron nitride and made into pellets. V-*K* edge spectra were measured by using a Si(111) double-crystal monochromator in transmission mode. Ionization chambers were used to measure the intensity of the incident and transmitted X-rays, and the quick scan technique (QXAFS) was used for this measurement. These spectra were normalized and analysed by using Athena^54^. Time-of-flight neutron diffraction (ND) experiments were conducted by using the total scattering spectrometer NOVA at the BL21 beamline of the Materials and Life Science Experimental Facility (MLF), Japan Proton Accelerator Research Complex (J-PARC)^55^. The samples were placed in a cylindrical vanadium cell (6 mm in diameter). The observed scattering intensities for the samples were corrected for instrumental background, absorption of samples and cell^56^, and multiple^57^ and incoherent scatterings and then normalized by the incident beam profile, which obtained from the scattering intensity for a vanadium rod. ^51^V spectra were measured at 11.7 T (JEOL, ECA-500 FT-NMR). A NaVO_3_ aqueous solution (0.16 mol dm^−3^) was taken as a reference (δ = −574.28 ppm). The pulse delay was 1 s. Raman spectra were recorded by using a LabRam spectrometer (Jobin-Yvon). A laser with an emission wavelength of 514.5 nm and a power of 0.2 mW was used.

### Structural modelling

The RMC modelling was performed on an ensemble of 3500, 4138, 3989 and 3818 particles for VP0, VP10, VP28 and VP44 glass, respectively. The starting configurations were generated by hard-sphere Monte Carlo simulations with constraints applied to avoid physically unrealistic structures. The constraints on the P-O connectivity were that all phosphorus atoms were coordinated for four oxygen atoms for atomic distances of up to 1.7 Å. X-ray *S*^X^(*Q*), neutron *S*^N^(*Q*) and *k*^3^*χ*(*k*) EXAFS data measured at the V-*K* edge were fitted simultaneously by using RMC + + code^58^. EXAFS back scattering tables were obtained from FEFF calculations^59^. The ring statistics were calculated by primitive rings analysis^60–62^ using R.I.N.G.S. code^63^. Cavity analysis was carried out by employing pyMolDyn code^64^. The cut-off distance *r*_c_ for cavity calculation was 2.3 Å.

## Supplementary information

Supplementary Information.

## Data Availability

The datasets generated during and/or analysed during the current study are available from the corresponding author upon reasonable request.

## References

[1] Zachariasen WH (1932). The atomic arrangement in glass. J. Am. Chem. Soc..

[2] Sun KH (1947). Fundamental condition of glass formation. J. Am. Chem. Soc..

[3] Mehrer, H. *Diffusion in solids: fundamentals, methods, materials, diffusion-controlled processes*, 522–523 (Springer, 2007).

[4] Denton EP, Rawson H, Stanworth JE (1954). Vanadate glass. Nature.

[5] Munakata M (1960). Electrical conductivity of high vanadium phosphate glass. Solid-State Electron.

[6] Linsley GS, Owen AE, Hayatee FM (1970). Electronic conduction in vanadium phosphate glasses. J. Non-Cryst. Solids.

[7] Frazier LL, France PW (1977). Compositional dependence of the electrical conductivity of vanadium phosphate glass. J. Phys. Chem. Solids.

[8] Roling B, Funke K (1997). Polaronic transport in vanadium phosphate glasses. J. Non-Cryst. Solids.

[9] Afyon, S. *et al*. New high capacity cathode materials for rechargeable Li-ion batteries: vanadate-borate glasses. *Sci. Rep*. **4**, 7113 (2014). 10.1038/srep07113 PMC5382707 25408200

[10] Uchaker E (2014). Better than crystalline: amorphous vanadium oxide for sodium-ion batteries. J. Mater. Chem. A.

[11] Arthur TS (2015). Amorphous V_2_O_5_-P_2_O_5_ as high-voltage cathodes for magnesium batteries. Chem. Commun..

[12] Aoyagi T (2016). Electrochemical properties and *in-situ* XAFS observation of Li_2_O-V_2_O_5_-P_2_O_5_-Fe_2_O_3_ quaternary-glass and crystallized-glass cathodes. J. Non-Cryst. Solids.

[13] Naito, T. *et al*. Lead-free low-melting and semiconductive vanadate glass applicable to low-temperature sealing. *Jpn, J. Appl. Phys*. **50**, 088002 (2011).

[14] Naito T (2013). Influence of P_2_O_5_/TeO_2_ composition ratio on the physical properties of V_2_O_5_-P_2_O_5_-TeO_2_ glasses for lead-free low-temperature sealing. J. Ceram. Soc. Jpn..

[15] Kubo S (2014). Characteristic evaluation of lead-free sealing glasses composed of V_2_O_5_-MnO_2_-KPO_3_-CuO. Kagaku Kogaku Ronbunshu.

[16] Matuo F (2015). Development of V_2_O_5_-ZnO-TeO_2_-(ZrO)_2_(HPO_4_)_2_ sealing glass with low melting point and low thermal expansion properties. Kagaku Kogaku Ronbunshu.

[17] Cho SJ, Lee K (2015). Additional study on the laser sealing of dye-sensitized solar-cell-panels using V_2_O_5_ and TeO_2_ containing glass. J. Korean Ceram. Soc..

[18] Li H (2012). Structure of V_2_O_5_-P_2_O_5_-Sb_2_O_3_-Bi_2_O_3_ glass. Int. J. Min. Met. Mater..

[19] Wang F (2012). Investigation of the melting characteristic, forming regularity and thermal behavior in lead-free V_2_O_5_-B_2_O_3_-TeO_2_ low temperature sealing glass. Mater. Lett..

[20] Feltz A, Unger B (1985). Redox reactions in condensed oxide systems II. Variation of the structure of vanadium phosphate glasses in dependence on the oxidation state of vanadium. J. Non-Cryst. Solids.

[21] Naito T, Namekawa T, Yamada S, Maeda K (1989). Effects of composition and additives on water durability in V_2_O_5_-P_2_O_5_ glass system. J. Ceram. Soc. Jpn..

[22] Sakurai Y, Yamaki J (1988). Correlation between microstructure and electrochemical behavior of amorphous V_2_O_5_-P_2_O_5_ in lithium cells. J. Electrochem. Soc..

[23] Khattak GD, Mekki A, Wenger LE (2009). X-ray photoelectron spectroscopy (XPS) and magnetic susceptibility studies of vanadium phosphate glasses. J. Non-Cryst. Solids.

[24] Hoppe U, Kranold R (1999). A reverse Monte Carlo study of the structure of vitreous V_2_O_5_. Solid State Commun.

[25] Hoppe U (2012). Structure of V_2_O_5_-P_2_O_5_ glasses by X-ray and neutron diffraction. J. Non-Cryst. Solids.

[26] Hoppe U, Walter G, Barz A, Stachel D, Hannon AC (1998). The P-O bond lengths in vitreous P_2_O_5_ probed by neutron diffraction with high real-space resolution. J. Phys. Condens. Matter.

[27] Salmon PS, Martin RA, Mason PE, Cuello GJ (2005). Topological versus chemical ordering in network glasses at intermediate and extended length scales. Nature.

[28] Kohara S, Suzuya K (2005). Intermediate-range order in vitreous SiO_2_ and GeO_2_. J. Phys. Condens. Matter.

[29] Hoppe U, Kranold R, Barz A, Stachel D, Neuefeind J (2000). The structure of vitreous P_2_O_5_ studied by high-energy X-ray diffraction. Solid State Commun.

[30] Munemura H, Tanaka S, Maruyama K, Misawa M (2002). Structural study of Li_2_O-V_2_O_5_ glasses by neutron and X-ray diffraction. J. Non-Cryst. Solids.

[31] Brazhkin VV (2011). Densified low-hygroscopic form of P2O5 glass. J. Mater. Chem..

[32] Eckert H, Wachs LE (1989). Solid-state 51V NMR structural studies on supported vanadium(V) oxide catalysts: vanadium oxide surface layers on alumina and titania supports. J. Phys. Chem..

[33] Lapina OB, Mastikhin VM, Simonova LG, Bulgakova YuO (1991). Characterization of surface species of supported V_2_O_5_-Al_2_O_3_ catalysts by 51V NMR. J. Mol. Catal..

[34] Miller JM, Lakshmi LJ (2000). V_2_O_5_ catalysts supported on Al_2_O_3_-SiO_2_ mixed oxide: 51V, 1H MAS solid-state NMR, DRIFTS and methanol oxidation studies. Appl. Catal. A.

[35] Nabavi M, Sanchez C, Livage J (1991). Structure and properties of amorphous V_2_O_5_. Philos. Mag. B.

[36] Sakida S, Hayakawa S, Yoko T (2000). 125Te and 51V static NMR study of V_2_O_5_-TeO_2_ glasses. J. Phys. Condens. Matter.

[37] Rozier P, Burian A, Cuello GJ (2005). Neutron and X-ray scattering studies of Li_2_O-TeO_2_-V_2_O_5_ glasses. J. Non-Cryst. Solids.

[38] Krins N (2007). Structural and electrical properties of tellurovanadate glasses containing Li_2_O. Solid State Ionics.

[39] Kohara S (2011). Relationship between topological order and glass forming ability in densely packed enstatite and forsterite composition glasses. Proc. Natl. Acad. Sci. USA.

[40] Akola J (2013). Network topology for the formation of solvated electrons in binary CaO–Al_2_O_3_ composition glasses. Proc. Natl. Acad. Sci. USA.

[41] Onodera, Y. *et al*. Formation of metallic cation-oxygen network for anomalous thermal expansion coefficients in binary phosphate glass, *Nat. Commun*. **8**, 15449 (2017). 10.1038/ncomms15449 PMC5499210 28561027

[42] Gharbi N (1981). A new vanadium pentoxide amorphous phase. J. Non-Cryst. Solids.

[43] Meyer K, Barz A, Stachel D (1995). Effects of atmospheric humidity on the infrared reflectivity of vitreous P2O5 and ultraphosphate glasses. J. Non-Cryst. Solids.

[44] Gin S (2013). An international initiative on long-term behaviour of high-level nuclear waste glass. Mater. Today.

[45] Cha J, Kubo T, Takebe H, Kuwabara M (2008). Compositional dependence of properties of SnO-P_2_O_5_ glasses. J. Ceram. Soc. Jpn..

[46] Fukui S, Sakida S, Benino Y, Nanba T (2012). Effect of Nb_2_O_5_ addition to SnO-P_2_O_5_ glass. J. Ceram. Soc. Jpn..

[47] Saitoh, A. *et al*. Zero photoelastic and water durable ZnO-SnO-P_2_O_5_-B_2_O_3_ glasses. *APL Mater*. **3**, 046102 (2015).

[48] Naito T, Namekawa T, Katoh A, Maeda K (1992). Effect of Sb_2_O_3_ addition on water durability of V_2_O_5_-P_2_O_5_ glass. J. Ceram. Soc. Jpn..

[49] Matunaga T (2011). From local structure to nanosecond recrystallization dynamics in AgInSbTe phase-change materials. Nat. Mater..

[50] Rosales-Sosa, G. A., Masuno, A., Higo, Y. & Inoue, H. Crack-resistant Al_2_O_3_-SiO_2_ glasses. *Sci. Rep*. **6**, 23620 (2016). 10.1038/srep23620 PMC4823710 27053006

[51] Drake CF, Stephan JA, Yates B (1978). The densities of V2O5/P2O5 glasses and the oxygen molar volume. J. Non-Cryst. Solids.

[52] Kohara, S. *et al*. Structural studies of disordered materials using high-energy X-ray diffraction from ambient extreme conditions. *J. Phys. Condens. Matter* **19**, 506101 (2007).

[53] Homma, T. *et al*. Full-automatic XAFS measurement system of the engineering science research II beamline BL14B2 at SPring-8. *AIP Conf. Proc.* **1234**, 13–16 (2010).

[54] Ravel B, Newville M (2005). ATHENA, ARTEMIS, HEPHAESTUS: data analysis for X-ray absorption spectroscopy using IFEFFIT. J. Synchrotron Radiat..

[55] Otomo, T. *et al.* Fundamental research of hydrogen storage mechanism with high-intensity total diffractometer. *KENS Rep.* **17**, 28–36 (2011).

[56] Paalman HH, Pings CJ (1965). Numerical evaluation of X-ray absorption factors for cylindrical samples and annular sample cells. J. Appl. Phys..

[57] Blech IA, Averbach BL (1965). Multiple scattering of neutrons in vanadium and copper. Phys. Rev..

[58] Gereben O, Jóvári P, Temleitner L, Pusztai L (2007). A new version of the RMC++ Reverse Monte Carlo programme, aimed at investigating the structure of covalent glasses. J. Optoelectron. Adv. Mater..

[59] Ankudinov AL, Ravel B, Rehr JJ, Conradson SD (1998). Real-space multiple-scattering calculation and interpretation of X-ray-absorption near-edge structure. Phys. Rev. B.

[60] Goetzke K, Klein HJ (1991). Properties and efficient algorithmic determination of different classes of rings in finite and infinite polyhedral networks. J. Non-Cryst. Solids.

[61] Yuan X, Cormack AN (2002). Efficient algorithm for primitive ring statistics in topological networks. Comp. Mater. Sci..

[62] Wooten F (2002). Structure, odd lines and topological entropy of disorder of amorphous silicon. Acta Cryst. A.

[63] Le Roux S, Jund P (2010). Ring statistics analysis of topological networks: new approach and application to amorphous GeS_2_ and SiO_2_ systems. Comput. Mater. Sci..

[64] Heimbach I (2017). pyMolDyn: identification, structure, and properties of cavities/vacancies in condensed matter and molecules. J. Comput. Chem..

