# Supplementary material for: Controlling oxygen coordination and valence of network forming cations
Source: Sci Rep. 2020 Apr 28;10:7178. doi: 10.1038/s41598-020-63786-y (PMC7188822; doi:10.1038/s41598-020-63786-y)
Supplement: Supplementary file 1 — Supplementary Information. [file 41598_2020_63786_MOESM1_ESM.docx]

Supplementary Information

Title

Controlling oxygen coordination and valence of network forming cations

Takuya Aoyagi, Shinji Kohara, Takashi Naito, Yohei Onodera, Motomune Kodama, Taigo Onodera, Daiko Takamatsu, Shuta Tahara, Osami Sakata, Tatsuya Miyake, Kentaro Suzuya, Koji Ohara, Takeshi Usuki, and Yamato Hayashi and Hirotsugu Takizawa

Figure S1 Normalized XANES spectra at V *K*-edge in VP*x* glasses. Absorption-energy at normalized intensity of 0.5 shifted to low energy as amount of P_2_O_5_ increased which means that vanadium was reduced by adding P_2_O_5_.

**Figure S2** Comparison between neutron/synchrotron X-ray data and RMC model for VP*x* glasses. **a**, Neutron total structure factor *S*^N^(*Q*). **b**, X-ray total structure factor *S*^X^(*Q*). **c**, EXAFS *k*^3^*χ*(*k*). EXAFS *k*^3^*χ*(*k*) data were obtained by back Fourier transformation of |FT(R)| of first correlation peak. Black curve, experimental data; Coloured curve, RMC model.

Figure S3 Raman spectra of VP*x* glasses. Peaks attributable to Q^2^ and Q^3^ sites appearing at more than 1150 cm^−1^ were not observed.

Figure S4 ^51^V MAS-NMR spectra of VP*x* glass samples.

Figure S5 Relationship between normalized weight loss in water and fraction of V^5+^O_4_ units in all cations.

Figure S6 Atomic packing density of VP*x* glasses. The density was calculated in accordance with literature^50^. Ionic radius of tetravalent vanadium in V^4+^O_4_ was assumed by linear approximation from ionic radius of V^4+^O_5_ and V^4+^O_6_ since it was not in data. Black point, calculated from literature data^24^; Blue point, experimental data.

Figure S7 Fraction of -M-O-ring distribution in VP*x* glasses. Purple, amorphous-VP0; Red, VP10 glass; Green, VP28 glass; Blue, VP44 glass.

Table S1 Fraction of V^5+^O_4_, V^5+^O_5_ and V^5+^O_6_ units in VP*x* glasses obtained with ^51^V MAS-NMR spectra.

| Composition | Fraction of V^5+^ structural units | | | |
| --- | --- | --- | --- | --- |
|  | VO_5_  (−300 ppm) | VO_6_  (−490 ppm) | *d*-VO_4_  (−540 ppm) | *s*-VO_4_  (−750 ppm) |
| VP10 | 0.27 | 0.08 | 0.12 | 0.53 |
| VP28 | 0.45 | 0.05 | 0.47 | 0.03 |
| VP44 | 0.81 | 0.10 | 0.08 | 0.02 |
